# Population Pharmacokinetics of Clozapine: A Systematic Review

**DOI:** 10.1155/2020/9872936

**Published:** 2020-01-07

**Authors:** Orwa Albitar, Sabariah Noor Harun, Hadzliana Zainal, Baharudin Ibrahim, Siti Maisharah Sheikh Ghadzi

**Affiliations:** School of Pharmaceutical Sciences, Universiti Sains Malaysia, 11800 USM, George Town, Penang, Malaysia

## Abstract

**Background and Objective:**

Clozapine is a second-generation antipsychotic drug that is considered the most effective treatment for refractory schizophrenia. Several clozapine population pharmacokinetic models have been introduced in the last decades. Thus, a systematic review was performed (i) to compare published pharmacokinetics models and (ii) to summarize and explore identified covariates influencing the clozapine pharmacokinetics models.

**Methods:**

A search of publications for population pharmacokinetic analyses of clozapine either in healthy volunteers or patients from inception to April 2019 was conducted in PubMed and SCOPUS databases. Reviews, methodology articles, in vitro and animal studies, and noncompartmental analysis were excluded.

**Results:**

Twelve studies were included in this review. Clozapine pharmacokinetics was described as one-compartment with first-order absorption and elimination in most of the studies. Significant interindividual variations of clozapine pharmacokinetic parameters were found in most of the included studies. Age, sex, smoking status, and cytochrome P450 1A2 were found to be the most common identified covariates affecting these parameters. External validation was only performed in one study to determine the predictive performance of the models.

**Conclusions:**

Large pharmacokinetic variability remains despite the inclusion of several covariates. This can be improved by including other potential factors such as genetic polymorphisms, metabolic factors, and significant drug-drug interactions in a well-designed population pharmacokinetic model in the future, taking into account the incorporation of larger sample size and more stringent sampling strategy. External validation should also be performed to the previously published models to compare their predictive performances.

## 1. Introduction

Clozapine is a tricyclic dibenzodiazepine antipsychotic drug that is commonly used in the treatment of schizophrenia, particularly in patients who are refractory or intolerant to the side effects of typical antipsychotics [1]^.^ As compared to other antipsychotic drugs, clozapine has less risk of undesired neurological effects and can even improve the negative symptoms to some extent [2]. Clozapine is the only second-generation antipsychotic drug approved to minimize the risk of suicide in patients with a history of schizophrenia [3]. However, due to the risk of agranulocytosis and other side effects, clozapine needs extensive blood levels monitoring [4]. Therapeutic drug monitoring (TDM) of clozapine is clinically relevant in certain situations, such as inadequate clinical response, signs of toxicity, onset of seizures, changes in concurrent medications, concurrent use of caffeine or smoking, concomitant liver disease, and suspected noncompliance [5].

Clozapine is metabolized by CYP1A2 and CYP3A4 enzymes in the liver to form norclozapine or N-desmethylclozapine, which is considered to be the major metabolite (20–30%) [6]. Norclozapine not only is a strong 5-HT_1C_ receptor antagonist but also has similar affinity to clozapine for D_2_ and 5-HT_2_ receptors [7]. Plasma clozapine levels are shown to be correlated with clinical effects. Nevertheless, due to its complex metabolism, there are significant inter- and intraindividual variations in clozapine serum levels for a given dose [8]. Factors affecting the clozapine serum levels reported vary significantly from study to study, and predictors of the variability are inconclusive. According to Perry's dosing nomogram, 47% of clozapine concentration variability were explained by dose, sex, and smoking status [9], while dose, sex, cigarette smoking, body weight, clozapine level, and clozapine : norclozapine ratio accounted for only 48% of the clozapine concentration variability in Rostami-Hodjegan nomogram [10].

Population pharmacokinetic modeling is extensively used to identify the pharmacokinetic parameters of a population and investigate the covariates that contribute to pharmacokinetic variability [11]. A few drug concentration measurements can guide dosage adjustments using the integration of the population pharmacokinetic model with the Bayesian forecasting method [12].

Over the last decades, several population pharmacokinetic studies on clozapine have been conducted. This review aimed to introduce a systematic comparison of the published clozapine population pharmacokinetic models as well as to explore identified covariates influencing the clozapine pharmacokinetics models which are yet to be explored.

## 2. Materials and Methods

### 2.1. Search Strategy

Data for this review were identified by systematic review of publications listed in PubMed and SCOPUS databases from inception to April 2019 using the following search terms: “clozapine” AND (“population pharmacokinetics” OR “pharmacometrics” OR “pharmacokinetic model” OR “popPK” OR “pop PK” OR “PPK” OR “nonlinear mixed effect model” OR “NONMEM” OR “bayesian”). Additional publications were identified by reviewing study reference lists and consulting expert review articles identified through the search.

### 2.2. Inclusion/Exclusion Criteria

**Figure 1:**
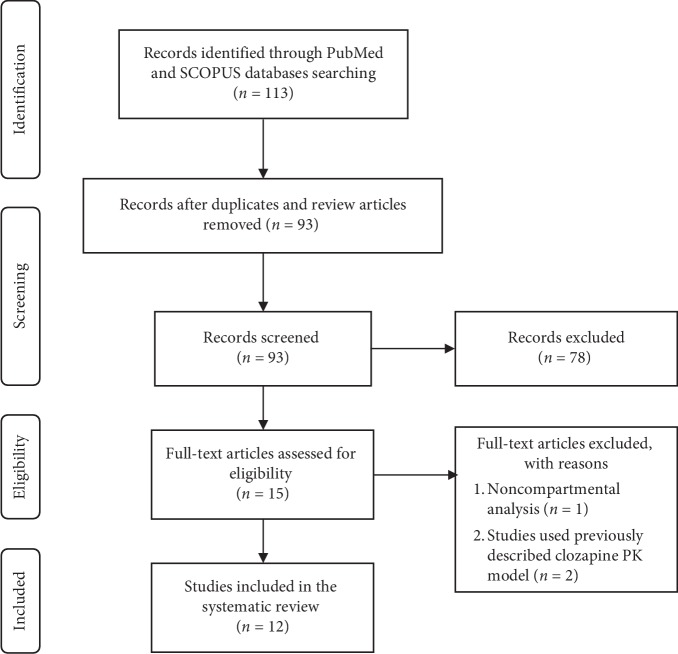
The selection process of the studies included in the systematic review.

The inclusion of studies was based on original studies describing population pharmacokinetic models for clozapine in healthy volunteers or in patients. Abstracts and other nonjournal publications were only included if sufficient details were provided. Reviews, methodology articles, in vitro and animal studies, and studies that used a previously described pharmacokinetic model as well as those involved noncompartmental analysis were excluded. The selection process is described in Figure 1 using the PRISMA 2009 flow diagram, which was previously described [13].

### 2.3. Data Extraction

Two independent reviewers extracted the relevant data from the included articles using a predesigned data collection form, and any disagreements were resolved by discussion. The variables that were retrieved from the identified studies include first author, publication year, country, number of subjects, subject characteristics (age, sex, weight, and pathology), clozapine dose, clozapine, and norclozapine levels, sampling schedule, and assay method, number of observations, observations per patient, data source, software used for modeling, structural and statistical model, tested and statistically significant covariates, and model validation which was further classified based on the increasing order of quality into three types: basic internal, advanced internal, and external model validation [14].

## 3. Results

### 3.1. Literature Search

The initial search strategy identified 113 potentially relevant citations, of which 93 remained after duplicates, and review-type articles were removed. After abstract and title scanning, 15 articles were retained for final evaluation. A total of 12 studies [15–26] published between 1987 and 2019 were included in this review, as demonstrated in Figure 1. Study characteristics of the included publications, samples, and concentrations are summarized in Table 1. The number of study participants varied from 13 to 391 (median: 130), totaling 1593 in all twelve publications with reported age ranging from 11 to 86 years. Only three of the studies had included subjects less than 18 years old [20–22]. The majority of the studies were in schizophrenia patients.

### 3.2. Analysis Methods Used in Pharmacokinetics Model Development

High-performance liquid chromatography (HPLC) was used to determine the serum levels of clozapine and norclozapine in all included studies except one study [16], where gas chromatography (GC) was used instead. The number of concentration readings (observations) ranged from 22 to 1617, with a median of 410, and the median observation per patient was 3. The daily dose was reported in nine studies with a median of 291 mg/day (134–540 mg/day) [15–19, 21, 22, 25, 26]. Most of the included studies used NONMEM software for population pharmacokinetic model [18, 20–26].

### 3.3. Structural Pharmacokinetics Model

The reported model structure, pharmacokinetic parameters, and covariates tested and retained in the final model are summarized in Table 2. In eleven of the included studies, the clozapine absorption was best described as first order [15, 16, 18–26]. The absorption rate was estimated in six of the included studies with a median of 0.69 h^−1^ (0.037–2.26 h^−1^) [16, 19–21, 25, 26], while it was fixed to a certain value from the literature in four of the studies [22–24, 26]. A one-compartment model was the best structural model that described the population pharmacokinetics of clozapine in most of the studies [16, 18–23, 25, 26], while the two-compartment model was reported in only three studies [15, 17, 24]. The median reported volume of distribution (Vd) was 508 L (272–1290 L) [15, 16, 18, 19, 21, 23] for clozapine and 624 L for norclozapine [23]. Elimination was best described as first order in six of the included studies [16, 19, 20, 22, 25, 26] with a median clearance (Cl) value of 30.3 L/h (14.4–45.2 L/h) for clozapine (*n* = 8) and 46.3 L/h (32.7–58.9 L/h) for norclozapine (*n* = 4). Model variability, error, and validation are summarized in Table 3. In eight of the studies, interindividual variability (IIV) was modeled using the exponential error model [18, 20–26]. The reported median IIV median (range) for clozapine Cl and Vd were 43.3% (27.1–60.8%) (*n* = 7) and 65.7% (10–131.5%) (*n* = 5), respectively, while for norclozapine Cl and Vd are 47.2% (42.1–60.25%) (*n* = 4) and 75.6% [23], respectively. The residual error was defined as proportional [18, 26], additive [25], or a combination of the two [20–24]. Model evaluation was performed in seven of the included studies [20–26] through either basic internal approaches such as goodness of fit [20, 24] and log-likelihood profiling (LLP) [22], or advanced internal approaches such as Jack-knife technique [21], normalized prediction distribution error (NPDE) [23, 24, 26], bootstrap [25, 26], visual predictive check (VPC) [25], and numerical predictive check (NPC) [26]. External evaluation using a validation group was performed in only one study [21].

### 3.4. Covariates

Several factors were tested in the modeling process, such as age, sex, CYP1A2 activity, weight, height, dose, smoking status, clozapine formulation, and coadministration of other drugs. Higher clozapine Cl was reported in smokers in five of the included studies [20, 21, 23, 24, 26], and in males in five of the reported studies [16, 20, 22–24], while sex failed to be included as a covariate in the final model of two studies [25, 26]. Age effect on clozapine Cl was controversial. In one of the included studies, the Cl has shown to be decreasing with increasing age [22]. However, no significant effect of age has been found for other studies [16, 18, 20, 23–26]. CYP1A2 was tested as a covariate in four of the studies included in this review paper [16, 18, 19, 21]. However, only two studies have found that CYP1A2 activity affects clozapine Cl [18, 19].

## 4. Discussion

Population pharmacokinetic modeling can be either parametric or nonparametric. The nonparametric makes no assumption regarding the shapes of the underlying parameter distributions, whereas parametric methods assume that the parameter and error distributions follow normal or log-normal distributions [27]. In this review, the authors focused on the parametric approach. The structural model developed in most of the studies was a one-compartment model with first-order absorption and elimination. Only six studies [15, 17, 18, 20, 22, 24] investigated the possibility of having a two-compartment model of clozapine. However, in only three studies, a two-compartment model was found to be the best fit for the data of clozapine [15, 17, 24]. These studies used a more stringent sampling strategy for not less than 12 hours. On the other hand, the studies that conclude a one-compartment model involved either a trough or random blood sampling schedule. Therefore, these studies may have less information needed to detect a two-compartment model of clozapine pharmacokinetics.

**Figure 2:**
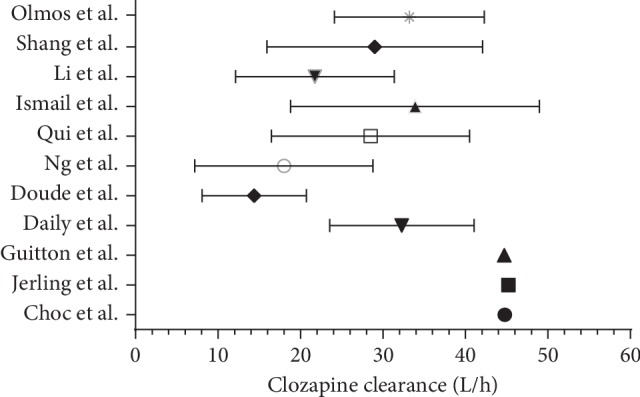
Clozapine clearance and between-subjects variability of the included studies.

Large intra- and interindividual variation in Cl and Vd was observed in most of the studies, as seen in Figure 2. This may be due to the intra- and interindividual variations in clozapine and norclozapine levels [28–30]. Many studies have demonstrated a correlation between the clinical response and the extent of clozapine conversion into norclozapine [31, 32]. However, norclozapine population pharmacokinetics was only evaluated in six of the included studies [17, 19, 20, 22, 23, 26]. Inadequate sample size was observed in many of the included studies limiting their power to detect covariate effects on PK parameters. It has been shown before that selection bias is very high for a small database of fewer than 50 subjects with weak covariate effects [33], and for a minimum of 3 observations, a sample size of more than 100 is recommended [34]. However, most of the studies have used only trough levels or a random level, which can lead to an inaccurate evaluation of the individual PK estimates especially the absorption rate constant. Furthermore, compliance assessment has not been carried out in most of the studies in order to make sure no changes in the clozapine dosing regimen had occurred before assessing the steady-state PK profile.

With regards to the covariates, smoking status was shown to be associated with higher clozapine Cl which leads to a lower concentration of clozapine among smokers [10, 30, 35–40]. This might be explained by the induction effect of cigarette smoking to the CYP1A2, which is the major metabolizing enzyme of clozapine [19, 41]. Nevertheless, smoking cessation has led to an increase in clozapine levels that result in toxicity development [42]. Norclozapine Cl was found to be higher in smokers [22]. Consistently, plasma levels of norclozapine have been reported to be lower in smokers as well [10, 35, 43]. However, these findings may be somewhat limited by the use of patient self-reporting to evaluate the smoking status in which the reliability of the report might be questioned. Furthermore, in all the studies, patients were classified into smokers and nonsmokers, not taking into account the magnitude of smoking or assessing an objective biological reading like serum nicotine or cotinine level. False-negative results of smoking status might lead to biased associations between the pharmacokinetic parameters and other less important factors.

Females had lower Cl of clozapine as compared to males, and this might be due to a lower CYP1A2 activity reported in females [44]. It has been demonstrated in most of the studies that females have significantly higher levels of clozapine, thus need a lower dose of clozapine as compared to men [10, 29, 35, 38, 40, 43, 45, 46]. Only a few studies have found no significant difference between males and females with regard to clozapine levels [9, 37, 39, 47]. Norclozapine Cl was found to be decreased in females as compared to males [22, 23]. Consistently, a higher concentration of norclozpine was observed in females [10, 29, 43, 45], while no difference in plasma levels was found in one study [46]. The possible reasons for the contradictory results may be related to insufficient or inequality in sample sizes, making the differences undetected.

The association between age and clozapine PK parameters was inconclusive between the studies. In one study, the age effect on clozapine PK was found to be significant [22]. It should be mentioned that this study has included the largest sample size (391 subjects) including elderlies, while other studies have found a negative association but had included a smaller sample size or not included elderlies; thus, they did not achieve sufficient power to detect such an effect. Age influence can be explained by changes in liver blood flow, size, or drug binding and distribution with advanced age [48]. Consistently, the association between age and clozapine plasma concentrations has been thoroughly demonstrated in the literature [10, 38, 45, 47, 49, 50]. These well-designed studies that involved a large sample size (more than 15000 samples [50]) and included elderly patients have achieved sufficient statistical power to present reliable results as compared to other less empowered studies that concluded a negative correlation [9, 29, 35]. Norclozapine Cl has shown to be decreased with the increasing age [22], which was consistent with higher norclozapine levels with increasing age [29, 43], while no correlation with age was found in another study [23].

Weight in the included population pharmacokinetic model had no effect on clozapine pharmacokinetics, in line with the finding from the published literature that observed no impact of weight on clozapine levels [45]. This was inconsistent with other studies that reported a higher clozapine concentration with increased weight [10] or body mass index (BMI) [46], as well as the tendency of deposition of clozapine in fat tissues that might eventually lead to a decrease in its Cl [51]. These findings were obtained with the use of only the total body weight (TBW) instead of other measures of body weight, such as adjusted body weight (ABW) and ideal body weight (IBW). Further investigation is needed to conclude the effect of weight on clozapine levels by incorporating different types of body weight such as TBW, ABW, and IBW.

The race effect has not been studied as a covariate on pharmacokinetic parameters of clozapine and should be considered in future studies. In one study, steady-state pharmacokinetics of clozapine between Maori and European patients was investigated and no significant difference in clozapine concentration was found [52]. Differences in metabolic phenotypes of cytochrome P450 enzymes were observed in different races/ethnicities [53]. As these enzymes are responsible for clozapine metabolism, the race could be a potential covariate explaining part of clozapine PK variability. Out of four studies that investigated the CYP1A2 activity as a covariate on clozapine pharmacokinetics, genotyping was performed only in one study, yet it still could not be included as a covariate [21]. In the remaining three studies, either clozapine/norclozapine ratio [18], caffeine test [19], or CYP1A2 distribution in other populations [16] were used to assess CYP1A2 activity. In literature, the majority of the studies have found significant effects of genetic variations in CYP1A2 on clozapine metabolism and plasma concentrations [30, 36, 54–57], as well as side effects [58]. CYP1A2 activity scores corrected for known inducers and inhibitors were associated with the dose-adjusted clozapine level [59]. Association could not be found in some studies [60, 61]. Only minimal effect of genetic variation of CYP3A4 on clozapine plasma levels or clinical response to clozapine was observed in the literature [54, 55], while in a recent study, a lower dose of clozapine was needed to achieve therapeutic levels in low CYP3A expresser [62]. Genetic polymorphism in uridine diphosphate glucuronosyltransferase (UGT) gene encoding to the enzyme responsible for clozapine glucuronidation was not found to be associated with clozapine plasma levels [54]. However, in one study, recent evidence was presented for associations of UGT polymorphisms and clozapine concentration [57]. Coadministration of valproic acid, antidepressant, antipsychotics, antidiabetics, and benzodiazepine was recently investigated in a population pharmacokinetic model but failed to be included as covariates [26]. However, the concomitant use of benzodiazepine was investigated even though only a pharmacodynamic interaction with clozapine was reported in the literature [63]. On the other hand, clozapine levels were found to be higher in patients receiving CYP1A2 inhibitors, such as fluvoxamine [39, 64, 65], CYP2D6 inhibitors as in paroxetine and fluoxetine [64], and CYP3A4 inhibitors like valproate [64, 66]. Ketoconazole (CYP3A4 inhibitor) impaired the clozapine N-oxide metabolite formation [65]. On the other hand, lower clozapine levels were observed with CYP3A4 inducers like phenobarbital [64] and carbamazepine [67]. Esomeprazole [59] and minocycline [68] have explained the variability in clozapine concentrations.

ATP-binding cassette (ABC) proteins have a principal role in hydrophilic compounds transportation through extracellular and intracellular membranes [69]. Genetic variation in ABCB1, known as P-glycoprotein 1 or multidrug resistance protein 1 (MDR 1) [55, 70], has correlated with clozapine levels and in a systematic review of genetic polymorphisms affecting clozapine PK in 2015, further studies were suggested to confirm this effect [71]. Recent studies have shown that ABCC1 [69] and ABCG2 [72] were found to affect clozapine concentration.

Metabolic profiling integration with pharmacokinetics identifies molecules than can be potential markers to predict pharmacokinetic variability and thus design an individualized drug regimen [73]. Many studies have investigated the association between metabolic profile and various drug pharmacokinetics. Clozapine shows to be a potential candidate for this kind of researches as it has been found in some studies that lipid dysregulation was associated with schizophrenia and lipid expression regulative patterns and other water-soluble metabolites can provide information related to the mechanism, side-effect, and potential target of antipsychotics [74, 75].

Population pharmacokinetic modeling requires careful selection and initial screening and evaluation of potential covariates based on scientific knowledge (pharmacology, biology, and pathophysiology) of the drug and the covariate of interest before proceeding to covariate model building [76, 77]. External model validation provides a more stringent assessment of models' predictive performance as compared to internal validation. Therefore, it is essential to be performed before the models can be applied in the clinical setting, for example, in the clozapine dose adjustment. However, external validation using a validation group was only performed in one study [21]; therefore, external validation of the previously published pharmacokinetics models should be conducted in the future to compare their predictive performances.

## 5. Conclusion

In conclusion, although several covariates were incorporated in the population models, the pharmacokinetic variability remained relatively large. Including other influential factors, such as genetic polymorphisms, metabolic factors, and potential drug-drug interactions, might explain the variability in a well-designed population pharmacokinetic model in the future, taking into account larger sample size and more stringent sampling strategy in order to be able to assess these factors efficiently. Besides, previously published models, as well as future models, should be evaluated externally for a more accurate description of models' performance.

## Figures and Tables

**Table 1:** Studies' population characteristics, samples, and concentrations.

Study	*N* (male/female)	Samples/subject	Total samples	Age (y^a^)	Body weight (kg^a^)	Site	Subjects characteristics	Dose (mg/day^a^)	Samples' time (h)	Clozapine concentration^a^ (ng/mL)	Norclozapine concentration^a^ (ng/mL)
Choc et al. [15]	13 (13/0)	33	429	34.2 ± 6.8	78 ± 11.7	USA and Georgia	Schizophrenia	150	0, 0.5, 1, 2, 4, 6, 9, 12	*C*_min_ = 142.1 *C*_max_ = 292.7	NA
Jerling et al. [16]	241 (159/82)	1.6	391	38 ± 10	NA	Sweden	Psychiatric patients	385	Random	*C*_*x*_ = 394.9	NA
Guitton et al. [17]	18 (13/5)	8	144	31 ± 9	70 ± 13.5	France	Schizophrenia	278	0, 0.5, 1, 2, 3, 5, 8, 12	*C*_min_ = 400.5 *C*_max_ = 914.5	*C*_min_ = 240 *C*_max_ = 377
Daily et al. [18]	23 (14/9)	3.6	83	37.6 ± 6.9	73.1 ± 14.7	France	Schizophrenia	540	Trough	NA	NA
Doude van Troostwijk et al. [19]	22 (19/4)	1	22	(28–64)	NA	Netherlands	Schizophrenia	426	Trough	*C*_0_ = 496 ± 220	*C*_0_ = 262 ± 107
Ng et al. [20]	197 (138/59)	2.6	519	38 ± 13	80.8 ± 18.5	Canada	Schizophrenia 98%	NA	Random	NA	NA
Qiu et al. [21]	183 (108/75)	3.4	626	49.7 ± 11.3	70.2 ± 11.9	China	Schizophrenia	289	Random	NA	NA
Ismail et al. [22]	391 (278/113)	2.9	1142	38.5 ± 12.4	82.5 ± 19.5	Canada	Schizophrenia	291	Random	*C*_*x*_ = 522.9 ± 326.8	*C*_*x*_ = 308.0 ± 172.4
Li et al. [23]	162 (74/88)	9.9	1617	35.5 ± 10.6	NA	China	Schizophrenia	NA	Random	*C*_*x*_ = 373 ± 239	*C*_*x*_ = 169 ± 100
Shang et al. [24]	198 (125/73)	7	1391	35 (18–59)	NA	China	Schizophrenia	(50–800)	Random	NA	NA
Li et al. [25]	47 (25/22)	3.3	154	37 (18–66)	67 (40–105)	China	Psychiatric patients	134	Trough	*C*_0_ = (309–4551)	NA
Olmos et al. [26]	98 (76/22)	1.7	171	39 [20–68]	78 [48–137]	Uruguay	Schizophrenia	350	Trough	*C*_0_ = 421 ± 262	*C*_0_ = 275 ± 180

*C*_min_: minimum concentration; *C*_max_: maximum concentration; *C*_*x*_: random concentration; *C*_0_: trough concentration; NA: not available. ^a^Values are mean, mean ± standard deviation, mean (range), or median [range].

**Table 2:** Model structure, pharmacokinetic parameters, and tested and retained covariates.

Study	Assay	Software	Structure model	Pharmacokinetic parameters		Covariates tested	Retained covariates in the final model
				Clozapine	Norclozapine		
Choc et al. [15]	HPLC	NONLIN	Two-compartment model with first-order absorption	Cl = 44.8 L/h Vd = 363 L F = 0.94	NA	NA	NA
Jerling et al. [16]	GC	NPML	One-compartment with first-order absorption and elimination	Cl = 45.2 L/h Vd = 666 L *K*_a_ = 0.096 h^−1^	NA	Age, sex, and, CYP1A2 activity	Lower Cl in females Lower Vd in females
Guitton et al. [17]	HPLC	NA	Two-compartment model	Cl = 44.7 L/h *V*_c_ = 7 L/kg *K*_10_ = 0.087 h^−1^ *K*_20_ = 0.156 h^−1^ *K*_12_ = 1.25 h^−1^	NA	NA	NA
Daily et al. [18]	HPLC	NONMEM	One-compartment model	Cl = 32.3 L/h Vd = 272 L	NA	Age, body weight, height, CYP1A2 activity, and daily dose	Higher Cl with higher CYP1A2 activity
Doude van Troostwijk et al. [19]	HPLC	MWPharm	One-compartment with first-order absorption and elimination	Vd = 4.3 L/kg F = 0.42 *K*_a_ = 0.98 h^−1^ Cl = 14.4 L/h *K*_e_ = 0.037 h^−1^	NA	CYP1A2 activity	Higher Cl with higher CYP1A2 activity
Ng et al. [20]	HPLC	NONMEM	One-compartment with first-order absorption and elimination	Cl = 18 L/h Vd^*∗*^ = 7 L/kg *K*_a_ = 0.14 h^−1^	Cl = 39 L/h	Age, sex, weight, smoking status, and dosage formulation	Higher Cl in smokers Lower Cl in females
Qiu et al. [21]	HPLC	NONMEM	One-compartment with first-order absorption	Cl = 28.5 L/h Vd = 1290 L *K*_a_ = 2.26 h^−1^	NA	Demographic index, coadministration of other drugs and CYP1A2 genotypes	Higher Cl in smokers
Ismail et al. [22]	HPLC	NONMEM	One-compartment with first-order absorption and elimination	Cl = 33.9 L/h Vd^*∗*^ = 950 L *K*_a_^*∗*^=0.8 h^−1^	Cl = 58.9 L/h	Age, sex, height, weight, and dosage formulation	Cl decreased with increased age Lower Cl in females
Li et al. [23]	HPLC	NONMEM	One-compartment with first-order absorption	Cl = 21.9 L/h Vd = 526 L *K*_a_^*∗*^=0.8 h^−1^	Cl = 32.7 L/h Vd = 624 L	Age, weight, sex, and smoking status	Higher Cl in smokers Lower Cl in females
Shang et al. [24]	HPLC	NONMEM	Two-compartment model with first-order absorption	Cl = 29 L/h *V*_c_ = 314 L *V*_p_ = 272 L *K*_a_^*∗*^=1.3 h^−1^	NA	Smoking, sex, age, and weight	Lower Cl in females Higher Cl in smokers
Li et al. [25]	HPLC	NONMEM	One-compartment with first-order absorption and elimination	Cl^*∗*^ = 21.9 L/h Vd^*∗*^ = 526 L *K*_a_ = 1.3 h^−1^ *K*_e_ = 0.0258 h^−1^	NA	Sex, hemoperfusion, age, toxic dosage, ratio of norclozapine to clozapine, trough concentration, and the time of gastric lavage	Lower *K*_e_ with higher reported intoxication dosage
Olmos et al. [26]	HPLC	NONMEM	One-compartment model	Vd^*∗*^ = 750 L *K*_a_ = 1.24 h^−1^ Cl = 32.1 L/h	Vd^*∗*^ = 1860 L Cl = 53.6 L/h	Smoking, age, sex, caffeine consumption, and coadministration of other drugs	Higher Cl in smokers

HPLC: high-performance liquid chromatography; GC: gas chromatography; NONMEM: nonlinear mixed-effects modeling; NONLIN: nonlinear regression; NPML: nonparametric maximum likelihood; Cl: clearance; Vd: volume of distribution; *V*_c_: volume of distribution of the central compartment; *V*_p_: volume of distribution of the peripheral compartment; *K*_a_: absorption rate constant; *K*_e_: elimination rate constant; *k*_10_: elimination rate constants from compartment 1; *k*_20_: elimination rate constants from compartment 2; *k*_12_: rate of metabolism; F: bioavailability; CYP: cytochrome P450; NA: not available. ^*∗*^Fixed value from the literature.

**Table 3:** Model variability, error, and validation.

Study	Interindividual variability		Error	Residual		Model evaluation
	Clozapine	Norclozapine		Clozapine	Norclozapine	
Daily et al. [18]	IIV Cl = 27.1% IIV Vd = 22.5%	NA	Pro	CV = 33.3%	NA	NA
Doude van Troostwijk et al. [19]	SD Vd = 1.01 L/kg SD F = 0.09 SD *K*_a_ = 0.01 h^−1^ SD Cl = 6.31 L/h SD *K*_e_ = 0.037 h^−1^	NA	NA	NA	NA	NA
Ng et al. [20]	IIV Cl = 60.8% IIV Vd = 131.5%	IIV Cl = 60.25%	Add + Pro	CV = 11.5%	CV = 9.5%	Basic internal (Goodness of fit)
Qiu et al. [21]	IIV Cl = 42.2% IIV Vd = 10%	NA	Add + Pro	SD = 45.8 ng/mL CV = 26.4%	NA	Advanced internal (Jack-knife) and external (validation group)
Ismail et al. [22]	IIV Cl = 44.5% IIV Vd = 93.2%	IIV Cl = 44.5%	Add + Pro	SD = 178.4 ng/mL	SD = 101.4 ng/mL	Basic internal (LLP)
Li et al. [23]	IIV Cl = 42.9% IIV Vd = 65.7%	IIV Cl = 42.1% IIV Vd = 75.6%	Add + Pro	SD = 52.9 ng/mL CV = 26.6%	SD = 36.6 ng/mL CV = 16.9%	Advanced internal (NPDE)
Shang et al. [24]	IIV Cl = 45.1% IIV *V*_c_ = 32.7% IIV *V*_p_ = 90.3% IIV *K*_*a*_^*∗*^=145.6 %	NA	Add + Pro	CV = 28.4%	NA	Basic and advanced internal (goodness of fit + NPDE)
Li et al. [25]	IIV *K*_e_ = 15.2%	NA	Add	SD = 0.149 h^−1^	NA	Advanced internal (bootstrap + VPC)
Olmos et al. [26]	IIV Cl = 43.3%	IIV Cl = 49.9%	Pro	CV = 9.54%	CV = 15.3%	Advanced internal (NPC + NPDE + bootstrap)

IIV: interindividual variability; CV: coefficient of variation; SD: standard deviation; Cl: clearance; Vd: volume of distribution; *V*_c_: volume of distribution of the central compartment; *V*_p_: volume of distribution of the peripheral compartment; *K*_a_: absorption rate constant; *K*_e_: elimination rate constant; F: bioavailability; Add: additive error; Pro: proportional error; LLP: log-likelihood profiling; NPC: numerical predictive check; NPDE: normalized prediction distribution error; VPC: visual predictive check; NA: not available. ^*∗*^Fixed value from the literature.
